# Neonatal Pseudohypoaldosteronism Type-1 in Japan

**DOI:** 10.3390/jcm11175135

**Published:** 2022-08-31

**Authors:** Kazumichi Fujioka, Ruka Nakasone, Kosuke Nishida, Mariko Ashina, Itsuko Sato, Kandai Nozu

**Affiliations:** 1 Department of Pediatrics, Kobe University Graduate School of Medicine, Kobe 650-0017, Japan; 2 Department of Pediatrics, Kakogawa Central City Hospital, Kakogawa 675-8611, Japan; 3 Department of Clinical Laboratory, Kobe University Hospital, Kobe 650-0017, Japan

**Keywords:** pseudohypoaldosteronism, neonate, aldosterone, renin, hyperkalemia, hyponatremia, mineralocorticoid receptor

## Abstract

(1) Background: Pseudohypoaldosteronism type 1 (PHA-1) is a disorder caused by renal tubular resistance to aldosterone and is characterized by problems with sodium regulation. PHA-1 is typically divided into primary PHA-1, which is caused by genetic mutation, and secondary PHA-1, which is associated with urinary tract abnormality. However, data on the clinical features of PHA-1 among newborn infants are limited. (2) Methods: We conducted a nationwide prospective surveillance study of neonatal PHA in Japan from 1 April 2019 to 31 March 2022 as part of a rare disease surveillance project of the Japan Society for Neonatal Health and Development. (3) Results: Fifteen cases (male:female = 7:8), including four primary, four secondary, and seven non-classified cases, were reported during the study period. The median gestational age and birthweight were 34 weeks (28–41) and 1852 g (516–4610), respectively. At the onset, the median serum Na and K levels were 132 mEq/L (117–137) and 6.3 mEq/L (4.7–8.3), respectively. The median plasma renin activity was 45 ng/mL/h (3.1–310, n = 9), active renin concentration was 1017 pg/mL (123–2909, n = 6), and serum aldosterone concentration was 5310 pg/mL (3250–43,700). (4) Conclusions: Neonatal PHA-1 was more common among preterm infants with no male predominance. It developed immediately after birth in cases without genetic or renal complications.

## 1. Introduction

Pseudohypoaldosteronism type 1 (PHA-1) is a disorder caused by renal tubular resistance to aldosterone and is characterized by hyperkalemia, hyponatremia, metabolic acidosis, and high renin and aldosterone concentrations [1]. In clinical settings, PHA-1 is a significant cause of neonatal hyperkalemia [2], and there are reports of life-threatening cases of excessive hyponatremia (113.3 mEq/L) associated with hyperkalemia (8.8 mEq/L) [3], and cardiac arrest due to excessive hyperkalemia [4]. PHA-1 is typically divided into the following two forms based on cause: primary PHA-1, which is caused by genetic mutation of the *NR3C2* gene encoding the mineralocorticoid receptor (MR) or of the epithelial sodium channel (*ENaC*) gene, and secondary PHA-1, which is associated with renal urinary tract abnormality. Primary PHA-1 is an autosomal dominant (*NR3C2*) or autosomal recessive (*ENaC*) hereditary disease that has been shown to block or inhibit regulation of MR in the target tissues [5]. Kuhnle et al. reported the transient reduction in lymphocytic MR in cases of secondary PHA-1 due to obstructive renal disease, with normalization after surgical correction of the obstruction [6]. The occurrence of secondary PHA-1 due to obstructive uropathies with and without infection during infancy and lateris well known. Kaninde et al. conducted a two-year prospective surveillance in Ireland and speculated the incidence of infantile secondary PHA-1 associated with urinary tract infection (UTI) and/or urinary tract malformation (UTM) to be 1 per 13,200 total live births per year [7].

Both primary and secondary PHA-1 include symptoms of sodium loss (aldosterone deficiency) from the neonatal period to infancy; however, they often do not require treatment after childhood [2,7,8]. It has been postulated that a developmental increase in tubular responsiveness to aldosterone might account for the improvement of PHA-1 [9]. In contrast, pseudohypoaldosteronism type 2, which is caused by genetic mutations in a family of serine-threonine kinases called with-no-lysine kinases (WNK) 1 and WNK4, results in hypertension, severe hyperkalemia, and mild metabolic acidosis in teenage patients [10].

Physiological renal tubular resistance to aldosterone has been suggested as the cause of PHA-1 predominance in the neonatal period. Martinerie et al. reported temporarily decreased mineral corticoid receptor gene expression at birth in the kidneys of mouse fetuses and newborns and decreased mineral corticoid receptor expression at 30–40 weeks’ gestation in the fetal kidney in experiments with human autopsy samples [11]. In addition, a prospective study of maternal and umbilical cord (neonatal) blood in non-complicated mother-child pairs revealed that the serum concentration of potassium, renin, and aldosterone was significantly higher in neonatal than maternal blood, suggesting partial physiological aldosterone resistance in human newborns in clinical settings [12].

However, data on the clinical features of PHA-1 among newborn infants in Japan are limited. Therefore, we conducted a nationwide survey of cases of neonatal PHA-1 in Japan.

## 2. Materials and Methods

A nationwide prospective surveillance study of neonatal PHA-1 was conducted from 1 April 2019 to 31 March 2022, which was part of a rare disease surveillance project of the Japan Society for Neonatal Health and Development (JSNHD). This study was approved by the institutional review board of Kobe University Graduate School of Medicine (approval number: B180252, 19 November 2018) and the Committee for Quality Improvement of the JSNHD. This study included neonates who showed both (1) symptoms of sodium loss (hyponatremia (Na < 135 mEq/L), the normal range in newborns; 135–145 mEq/L [13] and/or hyperkalemia (K > 6.0 mEq/L), the normal range in newborns; 3.5–6.0 mEq/L [14]) and (2) signs of renal tubular aldosterone resistance (hyperrenin-hyperaldosteroneemia: plasma renin activity > 10 ng/mL/h (average of healthy newborn infants on the 6th day; 1.5 ng/mL/h) [15] or active renin concentration > 100 pg/mL (normal range of adult; 2.21~39.49 pg/mL) and serum aldosterone > 3000 pg/mL (average of healthy newborn infants on the 6th day; 451 pg/mL) [15]). These cut-off values were based on the laboratory findings of three previously reported cases of PHA following ileostomy in preterm infants [16]. All members of the JSNHD can report their experience with PHA-1 on the JSNHD website. In addition, we included additional case reports obtained through personal communication between our pediatric nephrology laboratory (Prof. Kandai Nozu) and other institutes. Furthermore, a questionnaire on patient characteristics, including gestational age, birthweight, sex, Apgar scores, renal complications, and treatment, and laboratory data, including serum Na, K, renin, and aldosterone levels, and blood gas data at the onset, was sent to all participating members of the JSNHD that encountered PHA-1 cases. In addition, further detailed data on some cases were obtained by searching “Ichu-Shi Web”, a database provided by NPO Japan Medical Abstracts Society, using the terms “pseudohypoaldosteronism” and “neonate” between 2019 and 2022.

This study classified patients with confirmed genetic mutation in *NR3C2* or *ENaC* as primary PHA-1 and patients with renal or intestinal complications, excluding primary PHA-1, as secondary. Patients who fell into either of these two categories were categorized as exhibiting “classical PHA-1,” whereas patients that did not meet the criteria for either of the categories were categorized as “non-classified PHA-1”.

Data were expressed as medians (range) or numbers (percentages). Mann–Whitney U test, Chi-squared test, and Fisher’s exact test were used to compare the two groups. Differences were considered statistically significant at *p* < 0.05. Analyses were performed using GraphPad Prism version 7.00 (GraphPad Software, La Jolla, CA, USA).

## 3. Results

### 3.1. Primary Survey

From April 2019 to March 2022, 169 of 352 neonatal intensive care units (48%) covering 1727 of 3394 beds (51%) that were registered with the Ministry of Health, Labour and Welfare of Japan answered whether they experienced neonatal PHA-1 cases based on a survey conducted through the JSNHD website. During the study period, 24 cases were reported, including 12 cases registered from other institutes, 10 cases in our institute, and 2 personal communications between our pediatric nephrology laboratory (Prof. Kandai Nozu) and other institutes. The surveillance system missed the last two cases because they were not diagnosed in the NICU, but were later diagnosed with neonatal-onset PHA-1 at a pediatric nephrologist’s consultation. Nine cases were excluded; two cases were excluded due to type 2 PHA, confirmed by genetic examination (mutations in the CUL3 and KLHL3 genes), five cases were excluded due to laboratory data of either electrolytes or renin-aldosterone systems being within the range of reporting cut-off, and two cases were excluded due to the lack of a significant amount of data. One infant (Case 14) had low plasma renin activity but was included in this study, as primary PHA-1 was confirmed through the identification of the genetic mutation in NR3C2. Eventually, 15 cases (male:female = 7:8) were confirmed.

### 3.2. Secondary Survey

#### 3.2.1. Demographic Data

Demographic data are shown in Table 1. The median gestational age and birthweight were 34 weeks (28–41) and 1852 g (516–4610), respectively, and preterm infants accounted for 87% (13/15) of the sample. The Apgar scores were 8 (1–10) at 1 min and 9 (5–10) at 5 min. The mode of delivery was 10 emergency cesarean section (CS), 3 spontaneous vaginal deliveries, and 2 unknown. At disease onset, the median chronological age was 8 days (0–20), whereas the corrected age at disease onset was 34 weeks (30–42). There were several perinatal complications, including four twins (one monochorionic and three dichorionic; 27%), three cases who suffered from premature rupture of membrane (RPOM; 20%), three cases who suffered from non-reassuring fetal status (NRFS; 20%), three cases of gestational diabetes mellitus (GDM; 20%), and three cases of hypertensive disorders of pregnancy (HDP; 20%); conversely, three cases did not have any perinatal complications. In addition, there were several neonatal complications, including three cases born small for gestational age (SGA; 20%), two cases showing failure to thrive, which is known as a typical clinical symptom of PHA-1 (13%), and one case who suffered from cardiomyopathy and was later genetically diagnosed with cardiofaciocutaneous syndrome.

#### 3.2.2. Biochemical and Hormonal Profiles of Patients at Onset

The biochemical and hormonal profile of the patients are shown in Table 2.

At the onset, the median serum Na and K levels were 132 mEq/L (117–137) and 6.3 mEq/L (4.7–8.3), respectively. The median plasma renin activity was 45 ng/mL/h (3.1–310, n = 9), active renin concentration was 1017 pg/mL (123–2909, n = 6), and serum aldosterone concentration was 5310 pg/mL (3250–43,700). For renal function, blood urea nitrogen (BUN) and creatinine levels were 12.6mg/dl (5.1–40.8) and 0.69 mg/dL (0.28–1.18), respectively. For blood gas analysis, PH and base excess levels were 7.379 (7.25–7.558) and −1.9 (−13.1–2.3), respectively. Urinary Na data were available for only nine patients.

After the recovery, the median serum Na and K levels were 137 mEq/L (135–144) and 4.4 mEq/L (2.5–5.4), respectively. Although only limited data were available post-recovery, renin levels in 5 out of 6 (83%) available cases and aldosterone levels in 7 out of 7 (100%) cases decreased significantly.

#### 3.2.3. Therapy, Genetics, and Renal Data of the Patients

Therapy, genetics, and renal data of the patients are shown in Table 3. Regarding treatment, 12 out of 15 cases required Na supplementation and 2 cases required glucose-insulin therapy to control hyperkalemia. In 3 out of 15 cases, no treatment was required and spontaneous recovery was observed. The median chronological age of electrolyte recovery was 18 days (2–147). Causative factors, such as renal complications, included five cases of hydronephrosis and one case of renal hemorrhage. In addition, although only a limited number of cases was examined for genetic mutation, three patients had heterozygous mutation of *NR3C2* and one patient had homozygous mutation of SCNN1B; thus, those four cases were confirmed to be primary PHA-1.

#### 3.2.4. Comparison between Primary and Secondary PHA-1 and Others

We classified patients with confirmed genetic mutation as primary PHA-1 (Case 11, 12, 13, and 14), those with renal complications excluding primary PHA-1 as secondary (Case 1, 5, 9, and 15), and the rest as non-classified (Case 2, 3, 4, 6, 7, 8, and 10). The demographic data of the two groups (classical PHA-1 (primary and secondary PHA-1)) group vs. non-classified PHA-1 group) are shown in Table 4. There were no significant differences in the demographic and biochemical data between the two groups, although the incidence of emergency CS was relatively higher in the non-classified PHA-1 group and serum aldosterone levels, the incidence of Na supplementation, and postnatal days of electrolyte recovery were relatively higher in the primary and secondary PHA-1 group.

## 4. Discussion

This study clarified the clinical characteristics of PHA-1 in neonates. PHA-1 was common in preterm infants with no sex predominance and developed immediately after birth in some cases. In addition, this study revealed that some transient PHA-1 cases did not have any renal complications.

This nationwide prospective surveillance study in Japan is the first large-scale case series of PHA-1 among newborn infants. Ou et al. reported seven cases of newborns with secondary PHA-1 due to excessive gastrointestinal loss through high output stoma; however, the median onset of these cases was 39 postnatal days and the study only included one case (14%) of neonatal onset PHA-1 (<30 days). Thus, this does not reflect the clinical characteristics of PHA-1 in newborn infants. In addition, only 3 out of 7 cases had data on both renin and aldosterone levels at onset [17]. Kaninde et al. reported seven cases of transient infantile PHA-1 associated with urinary tract infection (UTI) and/or urinary tract malformation (UTM) from their prospective surveillance in Ireland. They concluded that the risk of transient PHA-1 was the greatest in boys with underlying urinary tract malformations, with or without concurrent urinary tract infection, within the first 3 months of life. Similar to the previous case series, only 2 out of 7 cases in their study had data on both renin and aldosterone levels at onset [7]. Likewise, Abraham et al. reported a case series of five patients with PHA-1 secondary to UTI from a single center retrospective study in Australia. In their study, all the patients were male and no correlation was apparent between plasma sodium and aldosterone levels in their PHA-1 populations [18]. As such, the present study has several strengths. It has the largest number of patients of any extant study, it rigidly targeted only newborn infants (<30 days of life), and it included all the electrolyte and hormone data at the onset. This study indicated that PHA-1 in newborn infants was more common in preterm infants, which is in line with a previous report [17]. Interestingly, Storey et al. reported that 68.6% of the patients that presented with hyponatremia under 100 days of age are preterm infants, and they proposed that those preterm infants have functional tubulopathy due to premature birth [19]. Thus, we may conclude that preterm infants are predisposed to developing PHA-1. Contrary to previous findings regarding infantile PHA-1, no male predominance was found in our neonatal PHA-1 cases [7,18].

In addition, we found that transient PHA-1 developed even in newborns without renal complications. In this regard, Storey et al. proposed the existence of transient aldosterone resistance in newborns without renal complications [19]. In general, the term “transient PHA-1” has often been used for secondary PHA caused by UTI and/or UTM [7,18]. However, our study revealed cases that met the biochemical and hormonal definition of PHA-1 very transiently, including having no renal complications. Interestingly, in Case 2, 3, and 7, electrolyte normalization was obtained without Na supplementation therapy within a very short period (within 5 days). In contrast, Na supplementation therapy was required in this study even in PHA-1 cases with unilateral hydronephrosis without infection, suggesting that renal complications should be considered an added factor to the requirement of therapy. This may suggest the presence of physiological aldosterone resistance of renal tubules in non-complicated preterm infants. Moreover, despite the lack of statistical significance between classical and non-classified PHA-1, all non-classified PHA-1 patients were preterm infants (<37 weeks of gestational age) with low birth weight (<2500 g), born via emergency CS associated with non-reassuring fetal status. An animal study using rat models of perinatal stress reported that the expression of mineralocorticoid receptors in neonatal-generated granule cells was downregulated by maternal daily restraint stress during late gestation [20]. Thus, fetal distress associated with premature birth may contribute to this postnatal renal tubular aldosterone resistance.

A limitation of the present study is that detailed follow-up data were not available, as this was a questionnaire survey. Moreover, not all cases in our population were genetically tested for *NR3C2* or *ENaC*. However, none of the cases in the non-classified group required Na supplementation for more than 2 months, which is significantly different from the typical course of primary PHA-1. Thus, we believe that the possibility of undiagnosed primary PHA-1 being included in our cases is low. At last, as there were cases in which patients with electrolyte disorders recovered without treatment within a very short period of time, there may have been a sufficient number of patients with undiagnosed transient PHA-1, who were not tested during the study periods. This hypothesis is based on the fact that more than half of all diagnosed cases were reported from our institute, which routinely conducts hormonal examination in cases of electrolyte disorders in newborns. Thus, to confirm the exact incidence of neonatal PHA-1 in Japan, a prospective study using a unified hormonal examination protocol for electrolyte disorders is required.

## 5. Conclusions

Neonatal PHA-1 was common in preterm infants with no male predominance in this study, and it developed immediately after birth in some cases, without any genetic or renal complications. For an accurate diagnosis of this disease, timely hormonal examination is required in cases of neonatal electrolyte disorders.

## Figures and Tables

**Table 1:** Demographic data of the patients.

Case	Gestational Age (Weeks)	Birth Body Weight (g)	Sex	AS 1/5	Mode of Delivery	Onset (Postnatal Days)	Perinatal Complication	Neonatal Complication
1	34	2562	F	5/7	SVD	1	PROM	Cardiofaciocutaneous syndrome
2	33	2436	F	8/9	ECS	1	PROM, HDP, GDM	None
3	30	1388	M	1/5	ECS	1	NRFS	Coarctation of the aorta, VSD
4	32	1604	F	7/8	ECS	12	Monochorionic twin	None
5	28	516	M	5/9	ECS	20	NRFS	SGA, PDA, Ascites
6	34	2000	F	7/9	ECS	1	Dichorionic twin	None
7	32	1692	F	5/7	ECS	1	Antiphospholipid syndrome, GDM	None
8	33	1852	M	8/9	ECS	12	PROM, dichorionic twin	None
9	33	1524	F	6/8	ECS	0	GDM, placenta previa	None
10	35	1572	M	8/9	ECS	13	HDP, dichorionic twin	SGA
11	36	3174	M	8/9	N/A	11	Schizophrenia	Failure to thrive
12	34	1562	F	8/10	ECS	10	HDP, NRFS	SGA
13	41	4610	M	8/9	SVD	8	None	Polycythemia
14	39	2698	F	9/10	SVD	5	None	Failure to thrive
15	34	2220	M	10/10	N/A	11	None	None

AS = Apgar scores; SVD = spontaneous vaginal delivery; ECS = emergency cesarean section; PROM = premature rupture of membrane; HDP = hypertensive disorders of pregnancy; GDM = gestational diabetes mellitus; NRFS = non-reassuring fetal status; VSD = ventricular septal defect; SGA = small for gestational age; PDA = patent ductus arteriosus; N/A = not applicable.

**Table 2:** Biochemical and hormonal profiles of the patients.

Case	Data at the Onset									Data Post-Recovery			
	Na (mEq/L)	K (mEq/L)	Renin (ng/mL/h or pg/mL)	Aldosterone (pg/mL)	BUN (mg/dL)	Creatinine (mg/dL)	pH	Base Excess (mmol/L)	Urine Na	Na (mEq/L)	K (mEq/L)	Renin (ng/mL/h or pg/mL)	Aldosterone (pg/mL)
1	124	5.7	21 (ng/mL/h)	3250	20	1.1	7.558	1.3	20	136	3.3	16.2 (ng/mL/h)	149
2	135	6.3	17.2 (ng/mL/h)	4300	9.8	0.85	7.343	−3.1	N/A	137	5.3	N/A	N/A
3	133	4.7	88.7 (ng/mL/h)	4800	10.7	0.72	7.449	−3.3	36	142	4.2	N/A	N/A
4	123	8.3	134 (pg/mL)	3900	12.6	0.89	7.397	−2.6	N/A	135	3.7	N/A	N/A
5	122	7	>45.0 (ng/mL/h)	7000	40.8	1.18	7.382	−1.9	N/A	141	2.5	>45 (ng/mL/h)	320
6	132	6.6	>45.0 (ng/mL/h)	5310	13.4	1.1	7.320	−1.6	N/A	144	4.7	N/A	N/A
7	129	6.1	2909.1 (pg/mL)	15,440.5	11.8	0.82	7.363	−5.4	24	136	4.3	N/A	N/A
8	133	5.7	619.21 (pg/mL)	3740.5	5.1	0.51	7.301	2.2	13	138	4.5	118.7 (pg/mL)	943.3
9	134	5.5	1416.21 (pg/mL)	4287.2	15	0.69	7.383	1.1	N/A	140	4.3	162.39 (pg/mL)	1185.1
10	137	6	263.82 (pg/mL)	3882.3	13.5	0.38	7.379	2.3	N/A	137	4.9	12.42 (pg/mL)	102.3
11	132	6.9	1415 (pg/mL)	39,165	20.7	0.68	7.354	−1.9	15	137	4.9	N/A	340
12	121	5.1	>20 (ng/mL/h)	18,100	5.4	0.5	7.397	0.8	18	139	N/A	N/A	N/A
13	128	7.9	310 (ng/mL/h)	26,400	11.1	0.28	7.250	−13.1	63	N/A	N/A	N/A	N/A
14	117	7	3.1 (ng/mL/h)	43,700	25	0.34	7.319	−3.1	15	N/A	N/A	N/A	N/A
15	134	6.4	245.9 (ng/mL/h)	7668	7.3	0.42	7.456	1.7	27.9	136	5.4	23.4	3423

N/A = not applicable.

**Table 3:** Therapy, genetics, and renal data of the patients.

Case	Treatment	End of Therapy (Postnatal Days)	Duration of Therapy (Days)	Electrolyte Recovery (Postnatal Days)	Renal Complication	Genetic Mutation
1	Na supplementation	183	182	47	Bil-hydronephrosis	N/A
2	None	None	None	2	None	N/A
3	None	None	None	5	None	N/A
4	Na supplementation, GI therapy	17	5	17	None	N/A
5	Na supplementation	21	1	31	Bil-hydronephrosis	N/A
6	Na supplementation	2	1	5	None	N/A
7	None	None	None	2	None	N/A
8	Na supplementation	22	10	26	None	N/A
9	Na supplementation	Continued	>400	7	Left renal Hemorrhage	None
10	Na supplementation	62	49	62	None	N/A
11	Na supplementation	N/A	>50	147	None	*NR3C2*
12	Na supplementation	N/A	N/A	18	Right-hydronephrosis	*NR3C2*
13	Na supplementation	N/A	>41	N/A	None	SCNN1B
14	Na supplementation, GI therapy	N/A	>7	N/A	Left-hydronephrosis	*NR3C2*
15	Na supplementation	22	11	33	Bil-hydronephrosis	N/A

Bil-Hydronephrosis = bilateral hydronephrosis; N/A = not applicable.

**Table 4:** Comparison of classical and non-classified PHA-1.

	Classical PHA-1 (n = 8)	Non-Classified PHA-1 (n = 7)	*p*-Value
Gestational age (weeks)	34 (28–41)	33 (30–35)	0.12
Birth body weight (g)	2391 (516–4610)	1692 (1388–2436)	0.40
Male sex	50% (4/8)	43% (3/7)	>0.99
AS 1	8 (1–10)	7 (1–8)	0.46
AS 5	9 (7–10)	9 (5–9)	0.16
Emergency cesarean section	50% (3/6)	100% (7/7)	0.07
Onset (postnatal days)	9 (0–20)	1 (1–13)	0.94
Na (mEq/L)	126 (117–134)	133 (123–137)	0.13
K (mEq/L)	6.7 (5.1–7.9)	6.1 (4.7–8.3)	0.63
Renin activity (ng/mL/h)	33 (3.1–310)	66.85 (17.2–134)	0.94
Renin (pg/mL)	1416 (1415–1416)	441.5 (134–2909)	0.53
Aldosterone (pg/mL)	12,884 (3250–43,700)	4300 (3741–15,441)	0.09
BUN (mg/dL)	17.5 (5.4–40.8)	11.8 (5.1–13.5)	0.19
Creatinine (mg/dL)	0.59 (0.28–1.18)	0.82 (0.38–1.1)	0.35
pH	7.383 (7.25–7.558)	7.363 (7.301–7.449)	0.56
Base excess (mmol/L)	−0.55 (−13.1–1.7)	−2.6 (−5.4–2.3)	0.80
Na supplementation	100% (8/8)	57% (4/7)	0.08
Duration of therapy > 1 week	86% (6/7)	50% (2/4)	0.49
Electrolyte recovery (postnatal days)	32 (7–147)	5 (2–62)	0.06

Classical PHA-1 included primary and secondary PHA-1.

## Data Availability

The data presented in this study are available in this article.
